# HA Antibody-Mediated FcγRIIIa Activity Is Both Dependent on FcR Engagement and Interactions between HA and Sialic Acids

**DOI:** 10.3389/fimmu.2016.00399

**Published:** 2016-09-29

**Authors:** Freek Cox, Ted Kwaks, Boerries Brandenburg, Martin H. Koldijk, Vincent Klaren, Bastiaan Smal, Hans J. W. M. Korse, Eric Geelen, Lisanne Tettero, David Zuijdgeest, Esther J. M. Stoop, Eirikur Saeland, Ronald Vogels, Robert H. E. Friesen, Wouter Koudstaal, Jaap Goudsmit

**Affiliations:** ^1^Janssen Prevention Center, Janssen Pharmaceutical Companies of Johnson & Johnson, Leiden, Netherlands

**Keywords:** hemagglutinin, Fc-receptor, stem-binding antibody, head-binding antibody, ADCC, CR9114, CR6261, CR8033

## Abstract

Interactions with receptors for the Fc region of IgG (FcγRs) have been shown to contribute to the *in vivo* protection against influenza A viruses provided by broadly neutralizing antibodies (bnAbs) that bind to the viral hemagglutinin (HA) stem. In particular, Fc-mediated antibody-dependent cellular cytotoxicity (ADCC) has been shown to contribute to protection by stem-binding bnAbs. Fc-mediated effector functions appear not to contribute to protection provided by strain-specific HA head-binding antibodies. We used a panel of anti-stem and anti-head influenza A and B monoclonal antibodies with identical human IgG1 Fc domains and investigated their ability to mediate ADCC-associated FcγRIIIa activation. Antibodies which do not interfere with sialic acid binding of HA can mediate FcγRIIIa activation. However, the FcγRIIIa activation was inhibited when a mutant HA, unable to bind sialic acids, was used. Antibodies which block sialic acid receptor interactions of HA interfered with FcγRIIIa activation. The inhibition of FcγRIIIa activation by HA head-binding and sialic acid receptor-blocking antibodies was confirmed in plasma samples of H5N1 vaccinated human subjects. Together, these results suggest that in addition to Fc–FcγR binding, interactions between HA and sialic acids on immune cells are required for optimal Fc-mediated effector functions by anti-HA antibodies.

## Introduction

Influenza viruses cause annual epidemics that affect 5–15% of the global population, resulting in approximately 3–5 million cases of severe illness and up to 500,000 deaths worldwide, particularly among the very young, the elderly, and the chronically ill (1–3). In addition, virus strains entering the human population from animal reservoirs occasionally give rise to pandemics increasing these numbers significantly (4).

Vaccination is considered the most effective way to lower influenza-related disease burden. Current influenza vaccines mainly aim to elicit antibodies that prevent the virus from interacting with the sialic acids on the host cells by targeting the head domain of hemagglutinin (HA), the major glycoprotein of the virus. However, since the head domain of the HA where the receptor-binding site is located evolves rapidly, influenza viruses often escape elimination by the immune system. Consequently, current influenza vaccines typically provide protection against narrow spectra of virus strains and must be updated regularly to match the circulating stains. Clearly, the development of a next generation of vaccines able to protect against all seasonal, as well as possible, pandemic strains, is a health-care priority.

Despite the diversity of the influenza HA, rare antibodies with broad *in vitro* neutralizing activity against influenza viruses [broadly neutralizing antibodies (bnAbs)] have been isolated from human memory B cells. In agreement with their *in vitro* activity, passive transfer of broadly neutralizing anti-influenza antibodies has been shown to protect mice and ferrets from lethal challenge with antigenically diverse viruses (5–12).

The structural characterization of several of these antibodies (5–7, 10–15) has revealed epitopes in the head and stem regions of the HA, where functional constraints appear to restrict the potential for the virus to mutate. These epitopes are of great interest as vaccine targets, and several strategies are being employed to generate vaccines that induce broadly reactive antibodies (16–18). To be able to effectively design these types of vaccines, it is essential to elucidate the underlying molecular mechanisms involved in the cross-protective immunity of these broadly reactive antibodies.

Influenza-specific antibodies can block essential steps in the viral life cycle. Depending on their epitope, they can directly interfere with the viral life cycle by blocking the binding of HA to its sialic acid receptors on the host cell, by preventing the low pH-induced conformational changes of HA required for membrane fusion, by inhibiting the cleavage of the HA0 precursor protein, or by inhibiting viral egress (5–7, 11, 13, 19, 20). Antibodies can also exert anti-viral effects through other mechanisms, including effector functions mediated by the Fc part of the antibody molecule, such as complement-dependent cytotoxicity (CDC) and antibody-dependent cellular cytotoxicity (ADCC) (21–24). Involvement of Fc-effector functions, in particular ADCC, has been demonstrated in the protection of mice from H1N1 challenge by bnAb FI6 (5).

Recent publications have shown that broadly reactive anti-HA head and stem antibodies require Fc receptor (FcR) engagement for optimal protection, while protection by strain-specific anti-HA head antibodies was independent of FcR interactions (25–28). In addition, it was shown that only stem-specific and broadly reactive anti-head antibodies, and not strain-specific anti-HA head antibodies, were able to engage FcγRs to trigger ADCC (25). No molecular mechanism to explain this observation has been proposed to date.

Here, we investigate the molecular mechanisms behind the observation that anti-stem antibodies and not anti-head antibodies are able to mediate robust FcγRIIIa activation. A panel of influenza A- and B-specific monoclonal antibodies with identical human IgG1 Fc domains, making them particularly suitable to compare their ability to mediate FcγRIIIa activation, were used. We demonstrate that in particular, anti-head antibodies that specifically inhibit the interactions between the HA receptor-binding site and sialic acids on immune cells fail to induce strong FcγRIIIa activation. The addition of such anti-head antibodies that block receptor binding can interfere with FcγRIIIa activation in human plasma. Based on our data, we propose a model that describes that optimal HA antibody-mediated FcγRIIIa activity is dependent on the interaction between HA on host cells and sialic acid receptors on immune cells.

## Results

### HAI-Positive Antibodies Are Unable to Induce Robust FcγRIIIa Activation

We have previously described broadly reactive antibodies that were protective *in vivo* against group 1 influenza A viruses (CR6261) (12, 13), antigenically diverse influenza B viruses (CR8033 and CR8071), and both group 1 and group 2 influenza A viruses as well as influenza B viruses (CR9114) (20). CR6261 binds the stem region of HA and neutralizes the virus by preventing the conformational changes of this protein that are required for the viral fusion process (12). In contrast, CR8033 and CR8071 bind non-overlapping epitopes in the head region of influenza B HA and neutralize *in vitro*. While CR8033 disrupts virus attachment to sialic acids on the surface of host cells – as can be measured by the hemagglutination inhibition (HAI) assay (HAI^+^) – CR8071 inhibits the release of progeny viruses from infected cells (19, 20). Interestingly, CR9114 binds to an epitope in the stem region of HA that is conserved among all influenza A and B viruses but only inhibits the viral fusion process of influenza A viruses and thus only has *in vitro* neutralizing activity against these viruses (20).

To further explore the molecular mechanism by which these broadly reactive antibodies provide protection *in vivo*, we characterized the antibodies, together with antibody 2D1, previously shown to inhibit the interaction between the HA and the sialic acids of several different H1N1 strains (29), in terms of their ability to induce effector functions as measured by a bio assay that measures antigen-specific antibody-dependent FcγRIIIa activation (30, 31).

**Figure 1:**
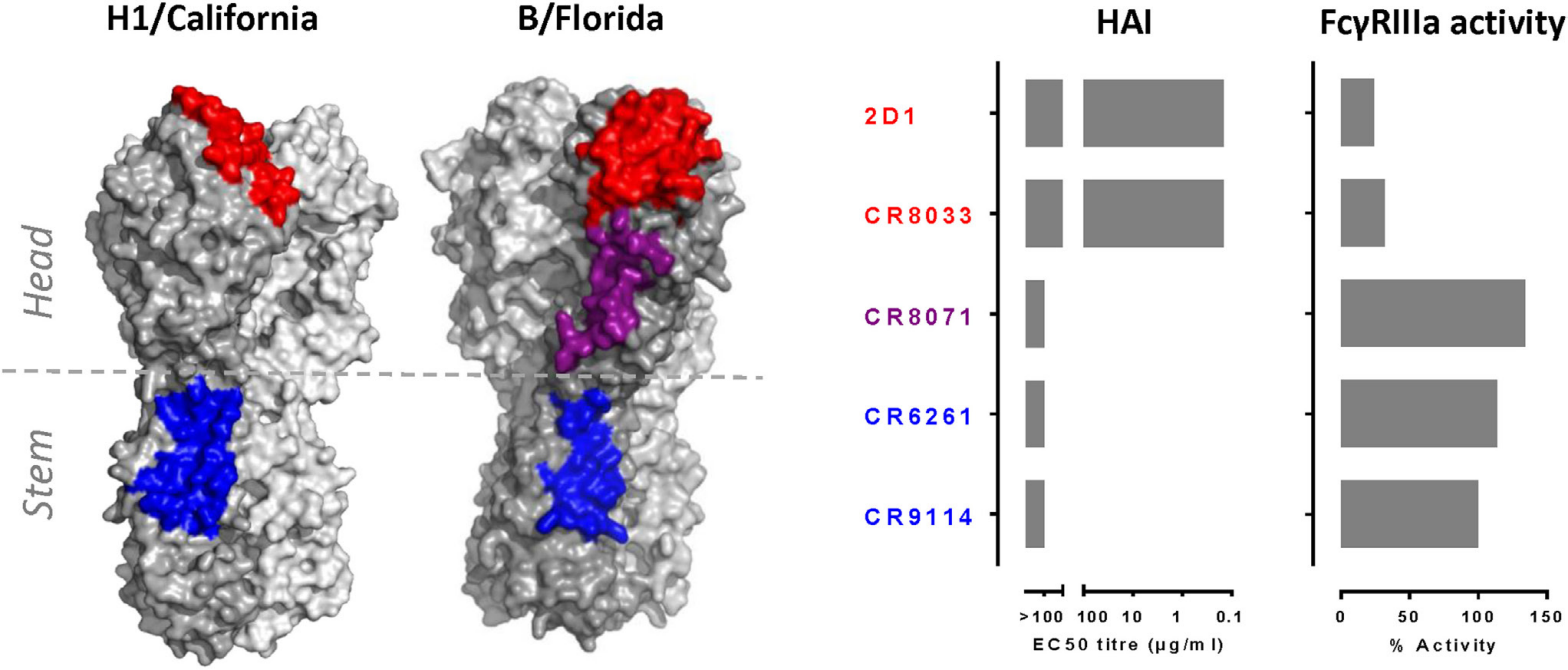
**HAI^+^ antibodies are unable to induce robust FcγRIIIa activation**. Left panel indicates approximate epitopes of 2D1 (in red), CR9114, and CR6261 (in blue) superimposed onto A/California/07/09 HA (H1/California) and approximate epitopes of CR8033 (in red), CR8071 (in purple), and CR9114 (in blue) superimposed onto B/Florida/4/2006 HA (B/Florida). Right panel indicates HAI and FcγRIIIa activation of antibodies 2D1, CR8033, CR8071, CR6261, and CR9114. HAI titers were measured for A/California/07/09 (2D1 and CR6261) or B/Florida/4/2006 (CR8033, CR8071, and CR9114). FcγRIIIa activation was measured using A/California/07/09 (2D1 and CR6261) or B/Florida/4/2006 (CR8033, CR8071, and CR9114) HA-transfected target human lung-derived A549 cells at an antibody concentration of 5 μg/ml. The FcγRIIIa activity was normalized against the response obtained with 5 μg/ml of CR9114. All antibodies contain identical human IgG1 Fc domains, making them particularly suitable to compare their ability to mediate FcγRIIIa activation. HAI and FcγRIIIa activity data are representative examples of three independent experiments.

The HAI^+^ antibodies CR8033 and 2D1 showed virtually no FcγRIIIa activation, while CR6261, CR8071, and CR9114 were able to induce robust FcγRIIIa activation to HA-transfected target cells (Figure 1).

### Sialic Acid Interactions Are Required for Optimal FcγRIIIa Activation

**Figure 2:**
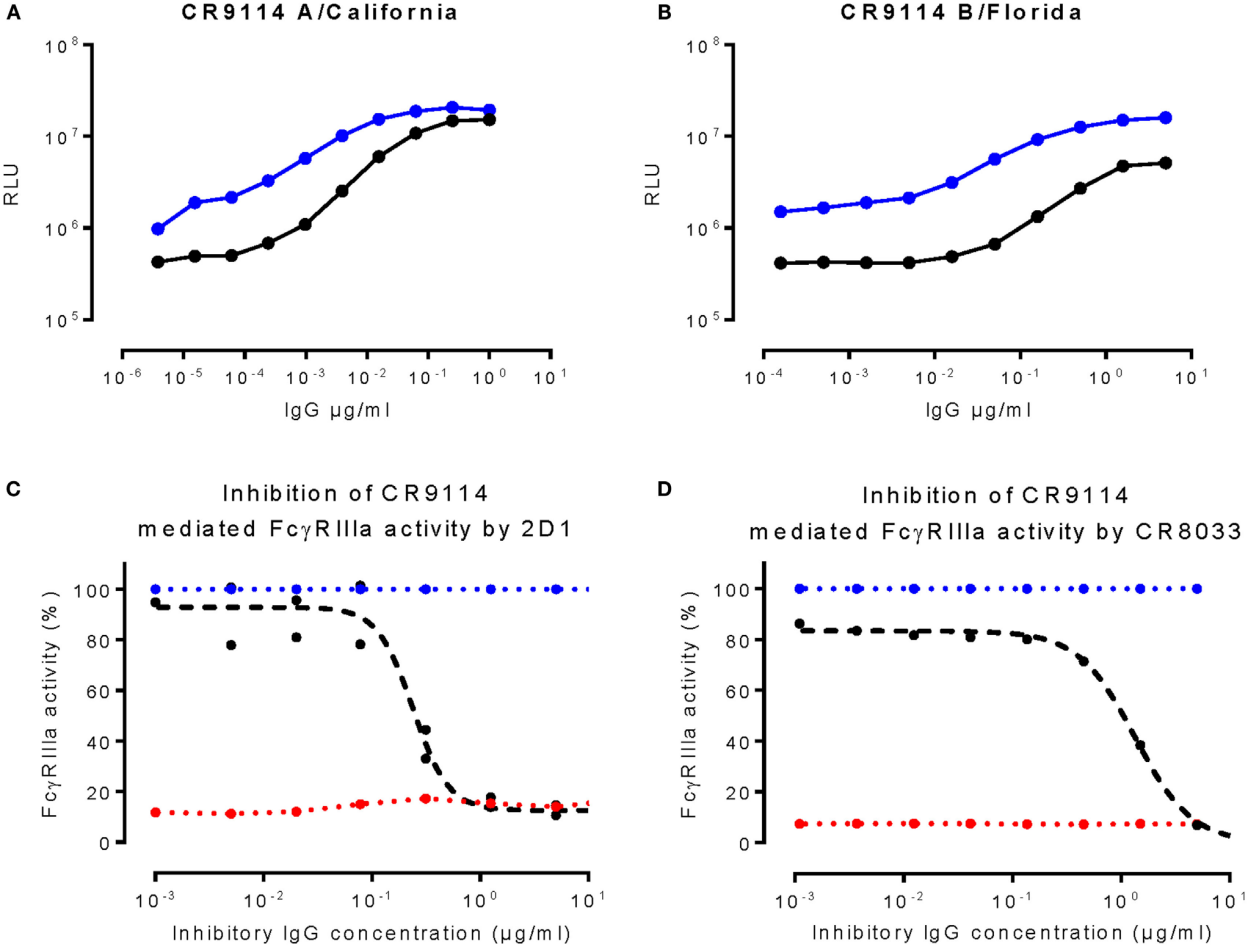
**Sialic acid interactions are required for optimal FcγRIIIa activation**. **(A,B)** depict FcγRIIIa activation against target cells transfected with a wild-type HA (blue) or non-SA binding mutant HA (black) of A/California/07/09 **(A)** or B/Florida/4/2006 **(B)**. **(C,D)** depict 2D1 or CR8033 (black curves) inhibiting FcγRIIIa activation against HA of A/California/07/09 **(C)** and B/Florida/04/06 **(D)**, respectively, induced by a fixed concentration (0.25 μg/ml) of CR9114. Background was determined by titrating in CR8033 or 2D1 to a non-binding antibody control (red dashed lines). As a control, a non-binding antibody was titrated in to the fixed concentrations of CR9114 (blue dashed line). RLU is the abbreviation for relative light units. Data presented are representative examples of at least three independent experiments.

The distinction between FcγRIIIa activation mediated by CR6261, CR9114, and CR8071 on one hand and CR8033 and 2D1 on the other appears to coincide with the ability to block HA–sialic acid interaction. This suggests that HA binding to sialic acid on immune cells may play a role in FcγRIIIa activation mediated by anti-HA antibodies. To investigate the role of HA–sialic acid interactions, a mutation in the HA of A/California/07/09 (L195F) and B/Florida/04/06 (L201F) was introduced preventing the HAs from interacting with sialic acid (non-SA binding HA) (Figure S1 in Supplementary Material). The FcγRIIIa activation to non-SA-binding HA mediated by CR9114 was much lower as compared to the FcγRIIIa activation to the wild-type HA (Figures 2A,B), while the antibody binding was comparable between the non-SA binding and wild-type HA (Figure S1 in Supplementary Material).

**Figure 3:**
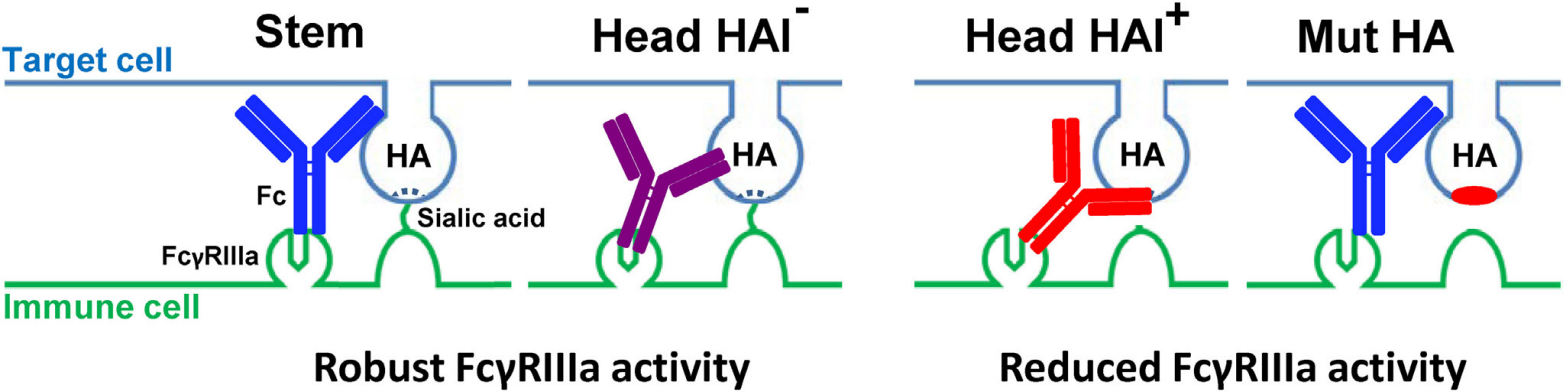
**Proposed model of differential effects of anti-stem (in blue), anti-head non-receptor blocking (HAI^−^) (in purple), and anti-head receptor blocking (HAI^+^) (in red) HA antibodies on target cell and immune cell interactions, using wild-type and non-SA-binding mutant HA, and the resultant effect on FcγRIIIa activation**.

The HAI^+^ antibodies 2D1 and CR8033 prevent the HA-expressing target cells from interacting with sialic acids on immune cells and may therefore be able to inhibit FcγRIIIa activation mediated by the HAI-negative antibodies CR6261, CR9114, and CR8071. In agreement with the dependency of potent FcγRIIIa activation on sialic acid interactions, 2D1 and CR8033 inhibit in a dose-dependent manner the FcγRIIIa activation induced by CR9114 binding to pH1N1 (A/California/07/09) HA and influenza B (B/Florida/04/06) HA, respectively (Figures 2C,D). Likewise, the FcγRIIIa activation mediated by CR6261 binding to pH1N1 HA and CR8071 binding to influenza B HA was inhibited by 2D1 and CR8033, respectively (Figure S2 in Supplementary Material). These results are consistent with a model in which interactions between HA and sialic acid residues on immune cells, in addition to the established antibody–FcγR interactions, are required for optimal FcγRIIIa activation by anti-influenza HA antibodies (Figure 3).

### The Presence of HAI^+^ Antibodies in Human Plasma Inhibits FcγRIIIa Activation

Upon exposure to influenza by infection or vaccination, humans develop both HAI^+^ and HAI^−^ antibodies (32). To assess whether the HAI^+^ antibodies inhibit FcγRIIIa activation in humans, we determined the HAI against H5N1 (A/Vietnam/1194/2004) and pH1N1 and FcγRIIIa activation in plasma from participants of a phase I dose-escalation study evaluating an H5N1 virosomal vaccine adjuvanted with Matrix-M (EudraCT no. 2008-006940-20). From the subjects who were vaccinated two times with a 30-μg HA dose of Matrix-M (50 μg/dose) adjuvanted virosomal vaccine, plasma samples were collected at day 0 (pre-vaccination), day 21 (post-prime), and day 42 (post-boost) for analysis. High levels of stem-binding antibodies were detected in these subjects after prime immunization. These levels were not boosted after the second immunization (Figure S4A in Supplementary Material).

**Figure 4:**
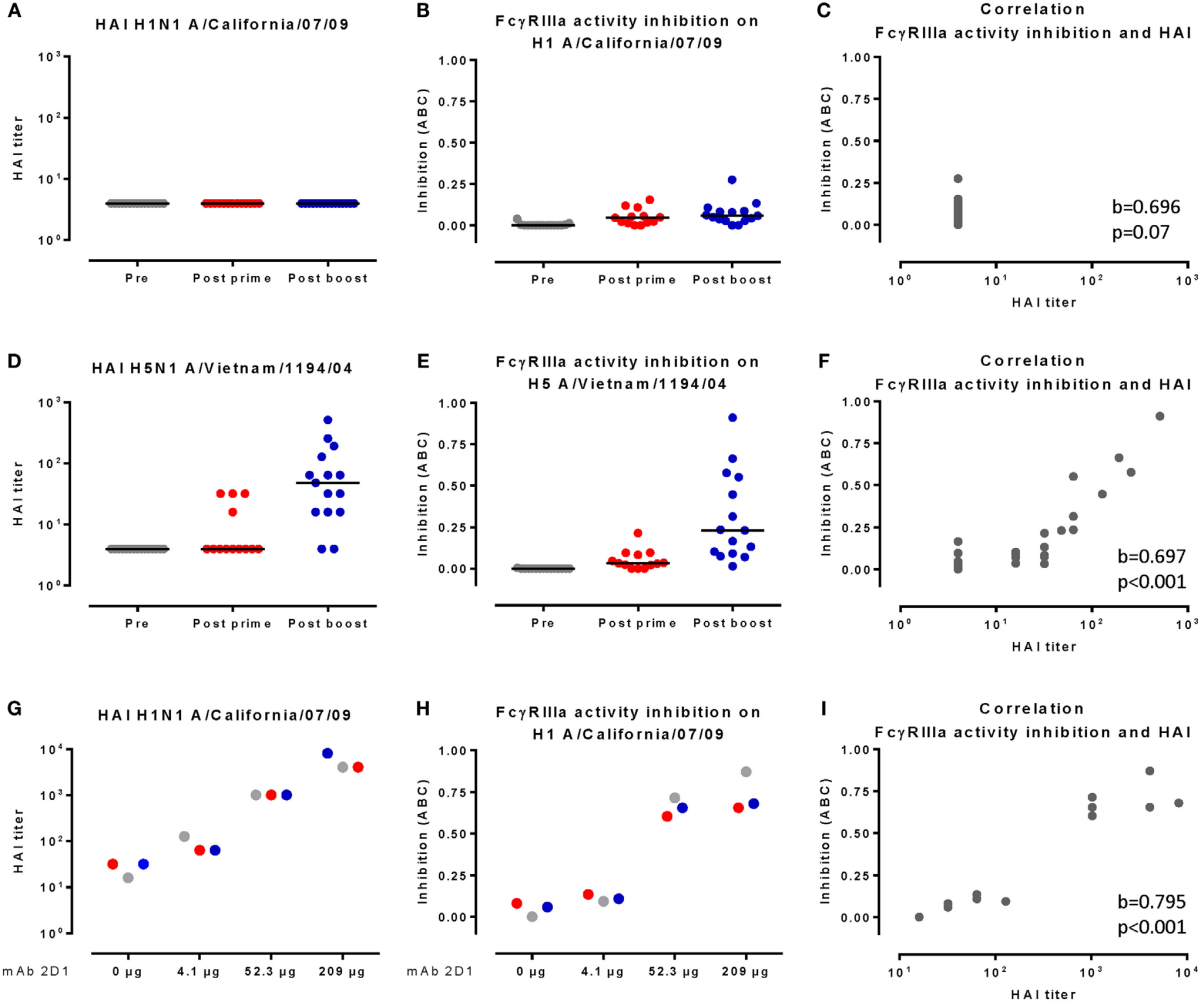
**HAI titers strongly correlate with inhibition of FcγRIIIa activity by human plasma**. **(A,D)** depict HAI responses of individual samples against pH1N1 **(A)** and H5N1 **(D)**. **(B,E)** depict pH1N1 HA-specific **(B)** and H5N1-specific **(E)** FcγRIIIa activation by individual samples. Black lines indicate the group median. **(G)** depicts the HAI response, and **(H)** depicts the pH1N1-specific FcγRIIIa activation inhibition of human plasma pools complemented with four different concentrations of mAb 2D1. Plasma samples taken before vaccination are indicated in gray (pre), 21 days after the prime in red (post-prime), and 21 days after the boost in blue (post-boost). The correlations between HAI responses and FcγRIIIa activation inhibition as determined by the Kendall’s tau-b method are depicted in **(C,F,I)**. The correlation is considered significant when *p* < 0.05. ABC indicates area between curves.

HAI^+^ antibodies against the vaccine homologous H5N1 strain, but not against the heterosubtypic pH1N1 strain, were induced, in particular, after the second vaccination (Figures 4A,D). In order to evaluate the inhibiting effect of HAI^+^ antibodies, we assessed the H5N1 HA-specific FcγRIIIa activation in plasma samples taken before (pre-vaccination) and after one (post-prime) and two (post-boost) vaccinations.

To quantify the amount of inhibition of FcγRIIIa activation, we used a modified four-parameter logistic curve (Eq. 2) to model the observed dose–response curve. Using this model, we estimated an inhibition corrected for the FcγRIIIa activation dose response and determined the area between the estimated and the observed dose–response curves as measure for FcγRIIIa activity inhibition (for an illustration, see Figure S3 in Supplementary Material).

H5N1 HA-specific FcγRIIIa activation was inhibited, in particular, in plasma taken after the boost immunization (Figure 4E). The inhibition of FcγRIIIa activation strongly correlated (Kendall’s tau b = 0.697, *p* < 0.001) with the HAI titers to H5N1 (Figure 4F). In line with our proposed model, pH1N1-specific FcγRIIIa activation in the absence of detectable HAI antibodies was barely inhibited (Figures 4A–C).

We next assessed pH1N1-specific FcγRIIIa activation in pools of individual plasma samples that were complemented with four different concentrations of the pH1N1 HAI^+^ antibody 2D1 or a control antibody. The levels of stem-binding antibodies were unaffected by the addition of 2D1 (Figure S4B in Supplementary Material), while both the HAI titers and the inhibition of FcγRIIIa activation increased in a 2D1 dose-dependent manner (Figures 4G,H). The levels of inhibition of FcγRIIIa activation and HAI titers were unaffected when control antibody was used (Figures S4C,D in Supplementary Material). The inhibition of FcγRIIIa activation strongly correlated with the HAI response against pH1N1 (Kendall’s tau b = 0.795, *p* < 0.001, Figure 4I), thereby confirming our results obtained with the monoclonal antibodies.

## Discussion

The identification and structural characterization of human antibodies with broad neutralizing activity against influenza viruses has raised hopes for the development of broad-spectrum antibody-based therapy and universal vaccines (33–37). Functional characterization of such bnAbs has revealed that depending on where they bind the HA molecule, they can directly interfere with the viral life cycle by blocking the binding of HA to its sialic acid receptors on the host cell, by preventing the low pH-induced conformational changes of HA required for membrane fusion, by inhibiting the cleavage of the HA0 precursor protein, or by inhibiting viral egress (5–7, 11, 13, 19, 20). In addition, effector functions mediated by the Fc part of the antibody molecule, such as CDC and ADCC (21), are involved in the protection against influenza as demonstrated in the protection of mice from H1N1 challenge by the bnAb FI6 (5).

Here, we use a bioassay to measure FcγRIIIa activation. FcγRIIIa is mainly expressed on human natural killer (NK) cells, and its activation leads to the release IFN-γ and cytotoxic mediators, such as perforin and granzyme that promotes cell death by triggering apoptosis (ADCC). The FcγRIIIa activation assay that we used correlates well with standard ADCC assays such as the classic PBMC-based ADCC assay as was determined by measuring the FcγRIIIa activation of different isotypes or fucosylation variants of human anti-CD20 and anti-EGFR antibodies (30, 38). FcγRIIIa in not exclusively expressed on NK cells but also on a subset of macrophages. Therefore, it may be that antibodies that induce FcγRIIIa activity also are able to induce other Fc-dependent effector function such as antibody-dependent cellular phagocytosis (ADCP).

Here, we show by analysis of a panel of monoclonal antibodies and human plasma samples from individuals vaccinated with an H5N1 vaccine that, in addition to FcγR interactions, interactions between HA on host cells and sialic acids on immune cells are required for optimal FcγRIIIa activation by anti-influenza HA antibodies.

Our finding could provide an explanation for recent results describing that FcγR interactions contribute to the *in vivo* efficacy of HA stem-binding, but not head-binding, antibodies against influenza A viruses (25, 26). In agreement with the previous observation, we found that the anti-head antibodies CR8033 and 2D1 did not efficiently induce ADCC *in vitro*, in contrast to the anti-stem antibodies CR6261 and CR9114. However, a robust FcγRIIIa activation was induced by another broadly reactive anti-head antibody, CR8071. The observation that not anti-HA head antibodies *per se*, but anti-HA head antibodies that block binding of HA to its sialic acid receptor, reduce FcγRIIIa activation led us to hypothesize that interactions between HA and sialic acid residues on immune cells, in addition to Fc–FcγR interaction, are needed to mediate optimal ADCC by anti-HA antibodies.

Cooperative binding of both cell-surface proteins (such as ICAM-1) and viral proteins (HA in the case of influenza) is known to play a role in the innate immune response to influenza; for example, it has been shown to be essential for NK cells to stimulate the formation of granules (39). This interaction between NK cells and HA was found to be dependent on sialylation of the NK cell receptor NKp46 (40), and NA-mediated removal of sialic acids from the NKp46 receptor on NK cells has been shown to constitute an immune-evasion mechanism of influenza viruses (41). In line with these findings, alterations in influenza viruses that lead to reduced affinity for sialic acid are associated with lower levels of innate NK cell activity (42).

It has been shown that immune complexes of anti-HA stem antibodies and HA are able to efficiently engage activating FcγRs, while immune complexes of an FcγR-blocking anti-HA head antibody and HA are not (25). However, an Fc point mutation that augments interactions with such activating FcγRs (GASD/ALIE mutant) restored FcγR binding, demonstrating that the Fc region of the anti-HA head antibody is accessible to FcγRs and not sterically hindered or spatially disrupted (25).

These findings are in line with, and explained by, a model in which Fc–FcγR interactions alone are not sufficient to efficiently mediate FcγR activation, and additional interactions between HA and sialic acids on the surface of immune cells are required (Figure 3) to reach high enough avidity between target and immune cell to trigger FcγR activation. They also suggest that lack of sialic acid binding can partially be overcome by mutations that increase the affinity of the antibody’s Fc domain for FcγRs.

We confirmed that the interactions between HA and sialic acid and between Fc and FcγR are both required for optimal FcγRIIIa activation by anti-influenza HA antibodies in polyclonal human plasma samples. Although these results endorse that FcγR activation is an important mechanism through which bnAbs provide protection against heterologous influenza strains, it is unclear what the contribution is against vaccine homologous strains, thus, in the presence of HAI^+^ antibodies. We have shown that HAI^+^ antibodies inhibit FcγRIIIa activation in human plasma and thereby are likely to reduce its contribution to protection. However, if the levels of HAI^+^ antibodies are high enough, they are able to prevent infection and effectively protect the host, independent from FcγRIIIa activation. Further studies are required to establish the implications for broad reactive influenza vaccines.

The fact that HAI^+^ antibodies inhibit FcγRIIIa activation in humans should be considered when accurate measurements of FcγRIIIa activation or ADCC activity are required.

Future vaccination strategies should take into account that – next to the induction of potent neutralizing antibodies – optimal effector functions may be an important hallmark of vaccine efficacy.

## Experimental Procedures

### Antibody Production

Human IgG1 antibodies, CR6261, CR9114, CR8033, and CR8071, were constructed by cloning the heavy (VH) and light (VL) chain variable regions into a single expression vector containing either the wild-type IgG1 constant regions or an L234A + L235A double mutant (for CR8033 LALA) that abolishes the ability of the Fc part of the antibody to interact with FcγR. The variable heavy and light chain of 2D1 (29) was also cloned into the same human IgG expression vector. The resulting IgG1 had identical specificity as the published antibodies (data not shown). The antibodies were produced on HEK293-F cells that were transfected with the IgG expression constructs, and the expressed antibodies were purified from serum-free culture supernatants using protein A chromatography (HiTrap, GE Healthcare, Buckinghamshire, United Kingdom) followed by a desalting step (HiPrep 26/10, GE Healthcare).

### Human Plasma Samples

Sixty healthy volunteers (22 males and 28 females) aged 20–49 years (mean age 31.2 years) were recruited into this clinical trial to evaluate a virosomal H5N1 vaccine that was performed between March and June 2009 at Haukeland University Hospital, Bergen, Norway. The open label phase I dose-escalating study was approved by the Regional Ethics Committee [Regional Committee for Medical Research Ethics, Northern Norway (REK Nord)] and the Norwegian Medicines Agency and registered in the European Clinical Trials Database (EudraCT no. 2008-006940-20). Informed consent was obtained from subjects prior to inclusion in the study. The subjects had never received an H5-based vaccine. Participants were randomly divided into four groups (*n* = 15) and vaccinated two times with 30-μg HA dose of non-adjuvanted vaccine or 1.5-μg HA, 7.5-μg HA, or 30-μg HA Matrix-M (50 μg/dose) adjuvanted virosomal vaccine, respectively, at days 0 and 21. At days 0, 21, 42, and for 6 and 12 months, blood samples were collected for analysis (43). For the current study, plasma samples collected at day 0 (unvaccinated; visit 1), 21 (after prime; visit 2), and 42 (after boost; visit 3) for the participants who received 30-μg HA adjuvanted with Matrix-M were used.

### Hemagglutination Inhibition Assay

Influenza virus H1N1 A/California/07/09 reassortant (NYMC X-181), B/Florida/04/06 (wild-type), or H5N1 A/Hong Kong/156/97 reassortant (four HA units per well) were combined in quadruplicate wells with an equal volume of antibody serially diluted in PBS. Plates were incubated for 1 h at 37°C in 96-well V-bottom plates. Fifty microliters of 1% turkey red blood cells was then added to each well and incubated for 1 h at room temperature. Button formation was scored as evidence of HAI. Titers were determined using the Spearman–Kärber formula.

### HA-Binding Assay

Binding of antibodies to wild-type and mutant influenza A and B HAs was tested by transfecting A549 cells with full-length recombinant HAs using Lipofectamin 2000 (Invitrogen, Carlsbad, CA, USA) to produce surface-expressed HA. On the following day, the cells were transferred to 96-well plates (PDL-coated black clear bottom) at a density of 1.5 × 10^4^ cells per well and incubated. Twenty-four hours later, cells were fixed (3% PFA, 15 min), washed, and incubated for 1 h with PBS/2%BSA at room temperature. Next, independent duplicates of serial antibody dilutions starting at 10 μg/ml were added to the cells (50 μl/well) and incubated for 3 h at room temperature, followed by wash steps and 30-min incubation with the secondary antibody [goat-anti-human IgG F(c) IRDye^®^ 800CW Conjugated] (Rockland, Limerick, ME, USA) diluted 1/500 in 5 PBS/2%BSA. After an additional wash step and fixation (1% PFA), fluorescence was measured on an Odyssey classic imager (Licor, Lincoln, NE, USA).

### CR9114 Competition ELISA

To determine stem-binding antibody responses, we measured CR9114-competing antibody responses as described before (44). Briefly, Maxisorp 96-well plates (Merck) were coated with purified polyclonal rabbit anti His-Tag IgG (Genscript, NJ, USA) O/N at 4°C followed by washing. After blocking and washing, plates were incubated with a titrated amount of His-Tagged HA of A/California/07/09 (in house produced and purified) for 2 h at RT. Plates were washed, and plasma was added to the plate in duplicate, serially diluted in block buffer, and incubated for 1 h at RT, followed by addition of a titrated amount of biotinylated human IgG1 CR9114 (produced and purified in house) and incubation for another hour at RT. After washing, streptavidin–HRP was added and incubated for 1 h at RT, followed by washing and OPD (Thermo Scientific, Bremen, Germany) development. The colorimetric reaction was stopped after 10 min by adding 1M H_2_SO_4_. The optical density (OD) was measured at 492 nm, and standard curves were created using a four-parameter logistic curve. The CR9114 competition of each sample was quantified as the slope of the linear regression of OD value on the log 10 dilution for the duplicate series.

### FcγRIIIa Activation Assay

Human lung carcinoma-derived A549 epithelial cells (ATCC CCL-185) were maintained in Dulbecco’s modified eagle medium (DMEM, Invitrogen) medium supplemented with 10% heat-inactivated fetal calf serum at 37°C. The A549 cells were transfected with wild-type B/Florida/04/2006 or A/California/07/09 (pH1N1) HA expression vectors or corresponding (non-sialic acid-binding mutants), using Lipofectamine 2000 (Invitrogen). The next day, 1.5 × 10^4^ A549 cells were seeded in white opaque flat bottom 96-wells plates. Eighteen hours later, antibodies or human plasma samples were serially diluted in assay buffer [4% ultra-low IgG FBS (Invitrogen) in RPMI 1640 (Invitrogen)]. Sample (antibody or human plasma) dilutions and ADCC Bioassay Jurkat effector (immune) cells expressing human FcγRIIIa and an NFAT-response element regulating a luciferase reporter gene (V158 variant provided by Promega, Fitchburg, MA, USA) were added to the cells and incubated for 6 h at 37°C. Cells were equilibrated at room temperature for 15 min before Bright-Glo luciferase substrate (Promega) was added. Luminescence was read out after 5 min on a Trilux Microbeta plate reader.

The interference experiments with monoclonal antibodies were performed in a similar fashion; however, instead of transfecting the A549 target cells with HA-expressing vectors, virus infection was used to obtain HA-expressing target cells. Therefore, 2 days before the experiment, 1.5 × 10^4^ A549 cells were seeded in white opaque flat bottom 96-well plates. One day before, the experiment cells were infected with 12,000 TCID_50_ per well influenza virus. The next day, independent duplicates of 2D1 or CR8033 LALA mutant (because wild-type CR8033 applied in very high concentrations did induce some FcγRIIIa activation) were eightfold serially diluted in assay buffer (4% ultra-low IgG FBS in RPMI 1640), and a fixed concentration of CR6261, CR9114, CR8071, or control antibody was added to each dilution. FcγRIIIa activity was normalized to the FcγRIIIa activity obtained with control antibody.

The interference by different concentrations of 2D1 or control antibody (4.1, 52.3, and 209 μg) in pooled plasma samples (an equal amount of the individual plasma samples per time point were pooled) was performed on A/California/07/09 (pH1N1) HA expression target cells as described above.

### Quantification of FcγRIIIa Activity Interference

FcγRIIIa activity dose–response curves are routinely fitted using a four-parameter logistic curve (Eq. 1). Here, the dose–response curve is summarized into four parameters: a lower asymptote (*A*), an upper asymptote (*D*), the inflection point (*C*), and a slope factor (*B*).

(1) $${\text{RLU}}_{\text{log}}=A+(D-A)\times \frac{1}{1+10^{(C-\text{log}X)\times B}}$$

which is equivalent to fitting a linear effect in logit-log space.

An inhibition of FcγRIIIa activity, reflected by a hook effect in the dose–response curve (Figure S4B in Supplementary Material, black dots), can be seen as a combination of an active and an opposite inhibition dose–response curve (Figure S4B in Supplementary Material, respectively, curves 1 and 2). When assuming that (i) the non-inhibited maximum FcγRIIIa activation (parameter *D*) and the minimum FcγRIIIa activation (parameter *A*) are shared between the two dose–response curves and that the two dose–response curves share a common (but opposite) slope factor, the four-parameter logistic curve can be modified to model 2 opposite linear effects simultaneously:

(2) $${\text{RLU}}_{\text{log}}=A+(D-A)\times \frac{1}{1+10^{(C-\text{log}X)\times B}}\times \frac{1}{1+10^{(IC-\text{log}X)\times -1\times B}}$$

where *B* represents the slope factor for the active dose–response curve, *C* and IC represents the inflection point of the active and the inhibiting dose–response curve, respectively (Figure S4B in Supplementary Material, curve 3).

For each subject, the amount of inhibition of FcγRIIIa activity at day 0 (unvaccinated; pre), at day 21 (post prime), and at day 42 (post boost) was determined by using Eq. 2 with time point-specific parameter estimates for *C*, IC, *B*, and *D*, whereas the estimate for *A* was shared between all three time points. In addition, to accommodate a possible vaccination effect on the FcγRIIIa activation (i.e., increase in FcγRIIIa activity at post prime or post boost), the model was constrained in such a way that the upper asymptote of the dose–response curves post prime or post boost had to be equal to, or higher than, the upper asymptote at pre-vaccination (i.e., dotted line in Figure S4A in Supplementary Material). For the data where antibody was spiked in a pool of sera, a similar approach was followed, without the constraint for the upper asymptote. The inhibition was then quantified as the area between the predicted (inhibition corrected) FcγRIIIa activity dose–response curve and observed dose–response curve (Figure S4C in Supplementary Material, area between, respectively, curves 1 and 3, depicted in green). Correlation between HAI titer and the amount of FcγRIIIa activity inhibition (i.e., area between curves) was assessed using Kendall’s tau-b.

### Generation of Influenza Pseudoparticles

Lentiviral pseudoparticles were generated by co-transfecting 293T cells using Lipo2000CD (Invitrogen) with pLP1, pLP2, and pLenti6-luciferase (Invitrogen), expression plasmids coding for influenza HA (wild-type and L201F mutant B/Florida/04/2006), neuraminidase (N1 HK/156/1997), and human airway trypsin-like (HAT, accession number NP_004253.1) (45). One day after the transfection, medium was replaced for culture medium (DMEM supplemented with l-glutamine, 5-mM MgCl, and 5% FBS; Invitrogen), and 96 h after transfection, the supernatants were passed through 0.45-μm filters and stored in aliquots at −80°C. HA content of pseudoparticles was determined by ELISA using anti-HA antibodies as described before (20).

### Fetuin ELISA

Maxisorp ELISA plates (Merck, Darmstadt, Germany) were coated overnight at 4°C with 30 μg/ml Fetuin (Merck) in PBS. Plates are blocked with 1% BSA in wash buffer (PBS + 0.05% Tween-20). Twofold dilutions of wild-type or L195F mutant A/California/07/09 HA or L201F mutant B/Florida/04/06 HA pseudotyped particles were added to the wells, and plates were incubated for 1.5 h at room temperature. Human CR9114 (2 μg/ml) and peroxidase-conjugated affinipure mouse anti-human IgG (Fcγ fragment specific, 1:1000; Jackson, West Grove, PA, USA) were used for detection. Plates were incubated with SigmaFast OPD (Merck) for 20 min; reactions were stopped by adding 1M H_2_SO_4_ and read using a Biotek plate reader.

## Author Contributions

FC and TK designed the studies and wrote the manuscript. BB, ES, RV, RF, and DZ assisted in designing the studies and provided essential scientific input. VK, BS, HK, EG, and LT performed and analyzed the studies. MK performed the statistical analysis. WK and JG managed the studies and assisted in writing the manuscript.

## Conflict of Interest Statement

All authors are or were employees of Janssen Research & Development, Janssen Pharmaceutical Companies of Johnson & Johnson.
