# Supplementary material for: HA Antibody-Mediated FcγRIIIa Activity Is Both Dependent on FcR Engagement and Interactions between HA and Sialic Acids
Source: Front Immunol. 2016 Sep 29;7:399. doi: 10.3389/fimmu.2016.00399 (PMC5040702; doi:10.3389/fimmu.2016.00399)
Supplement: Supplementary file 1 [file data_sheet_1.docx]

**Supplementary Figures**

**Supplementary Figure 1: Characterization of non-SA binding mutants.** Panel A and B: Binding curves of CR9114 on A549 cells expressing either wildtype A/California/07/09 (A) or B/Florida/04/06 (B) HA (blue) or L195F A/California/07/09 (A) or L201F B/Florida/04/06 (B) mutant HA (black). Error bars represent SD. Panel A and B: The ability of the wildtype and mutant HAs to interact with sialic acid was measured (n=1) in a Fetuin binding ELISA with pseudotype particles expressing either wildtype (blue) or mutant HAs (black). Per particle dilution a single measurement was performed.

**Supplementary Figure 2: 2D1 and CR8033 inhibit FcγRIIIa activation of CR6261, CR8071 or CR9114.** Upper Panels depict 2D1-mediated inhibition of A/California/07/09 HA specific FcγRIIIa activation (A) or CR8033-mediated inhibition of B/Florida/04/06 HA specific FcγRIIIa activation (B) induced by a fixed concentration (0.25 $\mu$g/ml) of CR6261 or CR8071, respectively (black curves). Background was determined by titrating in CR8033 or 2D1 mAb to a non-binding antibody control (red dashed lines). As a control, a non-binding antibody was titrated in to the fixed concentrations of CR6261 (green dashed line) orCR8071 (purple dashed line). Data presented are representative examples of at least 3 independent experiments.

**Supplementary Figure 3:** Illustration of the inhibition of FcγRIIIa activation calculation of the measured (black dots) of a subject to H5 expressing target cells pre-vaccination (A) or post boost (B and C) measured in duplicate. The inhibition was quantified as the area between the predicted (inhibition corrected) FcγRIIIa activity dose response curve and observed dose response curve (C, area between respectively curves 1 and 3, depicted in green).

**Supplementary Figure 4: Stem-binding antibodies are present in plasma of H5N1 vaccinated subjects and are unaffected by complementing 2D1.** Upper panel depicts the stem-binding antibody responses, as measured (n=2) by their ability to compete with CR9114 in individual (A) or pools of individual plasma samples (B). Black lines indicate median. Control antibody does not inhibit FcγRIIIa activation in human plasma. panel C depicts the HAI (n=1) and panel D depicts the inhibition of FcγRIIIa activity (n=1)) of pools of individual plasma samples complemented with 4 different concentrations of control antibody. Plasma samples taken before vaccination are indicated in gray, 21 day after the prime in red and 21 days after the boost in blue.
